# Comparison of non-Gaussian and Gaussian diffusion models of diffusion weighted imaging of rectal cancer at 3.0 T MRI

**DOI:** 10.1038/srep38782

**Published:** 2016-12-09

**Authors:** Guangwen Zhang, Shuangshuang Wang, Didi Wen, Jing Zhang, Xiaocheng Wei, Wanling Ma, Weiwei Zhao, Mian Wang, Guosheng Wu, Jinsong Zhang

**Affiliations:** 1 Department of Radiology, Xijing Hospital, Fourth Military Medical University, Xi’an, Shaanxi, P. R. China; 2 MR Research China, GE healthcare Greater China, Beijing, P. R. China; 3 Department of Gastrointestinal Surgery, Xijing Hospital, Fourth Military Medical University, Xi’an, Shaanxi, P. R. China

## Abstract

Water molecular diffusion *in vivo* tissue is much more complicated. We aimed to compare non-Gaussian diffusion models of diffusion-weighted imaging (DWI) including intra-voxel incoherent motion (IVIM), stretched-exponential model (SEM) and Gaussian diffusion model at 3.0 T MRI in patients with rectal cancer, and to determine the optimal model for investigating the water diffusion properties and characterization of rectal carcinoma. Fifty-nine consecutive patients with pathologically confirmed rectal adenocarcinoma underwent DWI with 16 b-values at a 3.0 T MRI system. DWI signals were fitted to the mono-exponential and non-Gaussian diffusion models (IVIM-mono, IVIM-bi and SEM) on primary tumor and adjacent normal rectal tissue. Parameters of standard apparent diffusion coefficient (ADC), slow- and fast-ADC, fraction of fast ADC (f), α value and distributed diffusion coefficient (DDC) were generated and compared between the tumor and normal tissues. The SEM exhibited the best fitting results of actual DWI signal in rectal cancer and the normal rectal wall (R^2^ = 0.998, 0.999 respectively). The DDC achieved relatively high area under the curve (AUC = 0.980) in differentiating tumor from normal rectal wall. Non-Gaussian diffusion models could assess tissue properties more accurately than the ADC derived Gaussian diffusion model. SEM may be used as a potential optimal model for characterization of rectal cancer.

Rectal cancer is one of the major causes of cancer-related mortality worldwide^1^. Early detection and accurate staging are important for the effective treatment of rectal cancer. Previous studies^2^,^3^,^4^,^5^,^6^,^7^,^8^ have demonstrated the values of DWI in diagnosis, prognosis and response to chemoradiotherapy of rectal cancer. So far, an apparent diffusion coefficient (ADC) value is effective in judging the outcome of chemoradiotherapy in rectal carcinoma and it has been widely accepted in clinical study that an increase of the value will demonstrate a positive therapeutic efficacy of rectal cancer^9^,^10^,^11^,^12^,^13^. However, in predicting tumor staging, as well as histological differentiation grades and mesorectal fascia (MRF) status, there has been no consistent conclusion in the previous findings regarding ADC value. Sun *et al*.^4^ showed that mean tumor ADC significantly differed between groups stratified by T stage, but it did not differ in the studies by Akashi *et al*.^8^ and Curvo-Semedo *et al*.^2^. This may be attributed to the following reasons^7^. Firstly, the measurement variation of ADC (including the observer’ various preferences for ROI selection^3^, low signal to noise ratio (SNR) at high b values, geometrical distortions caused by intra-rectal air^14^ and DW-MRI acquisition protocol). Secondly, the above conclusions were based on Gaussian diffusion behavior (the mono-exponential model) which presumed water diffusion behavior as free diffusion (Brownian diffusion) and simply described as average diffusion value. However, water molecular diffusion *in vivo* tissue is much more complicated and always presents non-Gaussian behavior^15^.

So a Non-Gaussian model was proposed to more closely reflect and evaluate the distribution of water molecular diffusion in human tissue, thus to profile the more realistic physiologic and pathologic characteristics of vivo tissue, such as cellularity, microcirculation and heterogeneity^16^. Nowadays, there are many non-Gaussian diffusion models to analyze DWI data, such as IVIM, and SEM. Each model was put forward to take non-Gaussian diffusion of water through tissue into account and to more comprehensively describe biological tissue properties by using different parameters.

Up to now, there has been much research in regard to the applications of non-Gaussian diffusion models in the nervous system, liver and prostate^17^,^18^,^19^,^20^,^21^, but such studies are rare in relation to rectal cancer. In the present study, with the purpose of choosing an effective diffusion model to be used in future research in rectum neoplasm, we aim to find out which model can fit the characteristics of water diffusion in rectal tumors and normal tissue better and to make a preliminary assessment of the robust functional parameters derived from the corresponding diffusion model.

## Result

### Repeatability measurements between inter-observers

The intraclass correlation coefficients (ICCs) for all parameters (Standard ADC, slow- and fast-ADC, f, α and DDC) were higher than 0.75 (P < 0.01, for each parameter), which showed good measurement reliability of quantitative MRI parameters.

### Model fitting test

**Figure 1:**
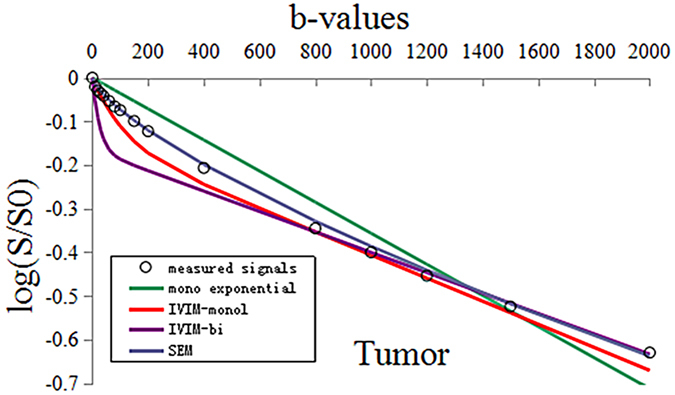
The measured DWI signals and fitting curves of the tumor. The stretched-exponential model (R^2^ = 0.998) achieved significantly better fitting of tumor DWI signal decay than others [the mono-exponential model (R^2^ = 0.937), the IVIM-mono model (R^2^ = 0.968), and the IVIM-bi model (R^2^ = 0.703)].

**Figure 2:**
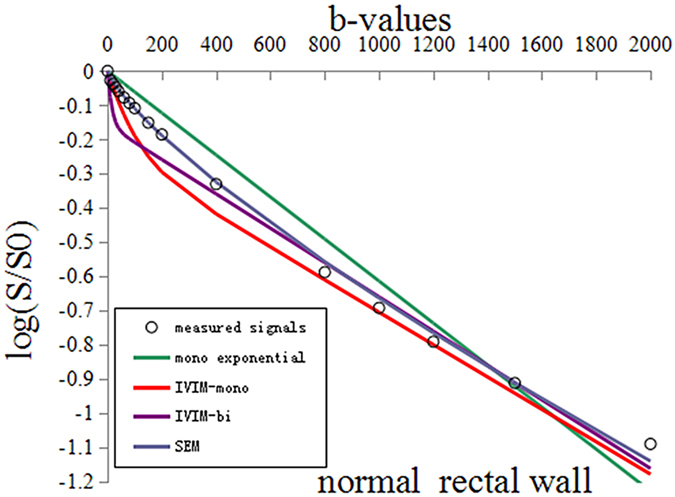
The measured DWI signals and fitting curves of the normal rectal wall. The stretched-exponential model (R^2^ = 0.999) achieved significantly better fitting of normal rectal DWI signal decay than others [the mono-exponential model (R^2^ = 0.952), the IVIM-mono model (R^2^ = 0.929), and the IVIM-bi model (R^2^ = 0.830)].

We fitted the different models with the average DWI signals of whole subjects on tumor and normal, respectively. As seen in Figs 1 and 2, the actual diffusion attenuated signals demonstrated substantial deviation from the mono-exponential attenuation, reflecting non-Gaussian diffusion behavior in rectal cancer and the normal tissue. For cancer tissue, the R^2^ value was 0.998 (P < 0.01), 0.937 (P < 0.01), 0.968 (P < 0.01), and 0.703 (P < 0.01) in SEM model, mono-exponential model, IVIM-mono model and IVIM-bi model, respectively. For normal tissue, the R^2^ value was 0.999 (P < 0.01), 0.952 (P < 0.01), 0.929 (P < 0.01), and 0.830 (P < 0.01), in SEM model, mono-exponential model, IVIM-mono model and IVIM-bi model, respectively. More specifically, compared to the Gaussian diffusion model, the SEM and IVIM-mono models achieved a significantly better fitting of tumor DWI signal decay while the IVIM-bi model resulted only in a comparably good fit in normal tissue but a poor fit in rectal cancer.

### Functional parameters between tumor and normal tissues

**Figure 3:**
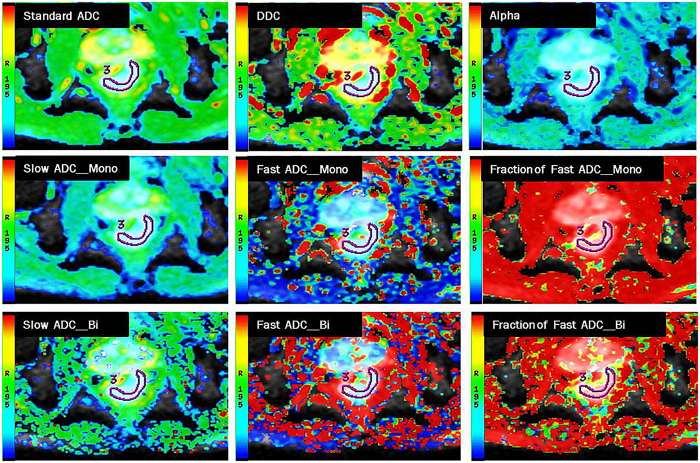
The different functional parameter pseudo color images of a 56 years old male patient with moderately differentiated rectal adenocarcinoma. ROI 3 represents the tumor area.

DDC and α value derived from SEM showed statistical difference between tumor and the normal rectal wall. The statistical differences were also found in slow-ADC of IVIM-mono and IVIM-bi, fast-ADC of IVIM-bi, and standard ADC of the mono-exponential model (all P < 0.001). The coefficient of variation (CV) of fast ADC-bi was significantly higher than that of other parameters. Detailed results are shown in Table 1 and the functional parameter map is shown in Fig. 3.

### ROC analysis of functional parameters

**Figure 4:**
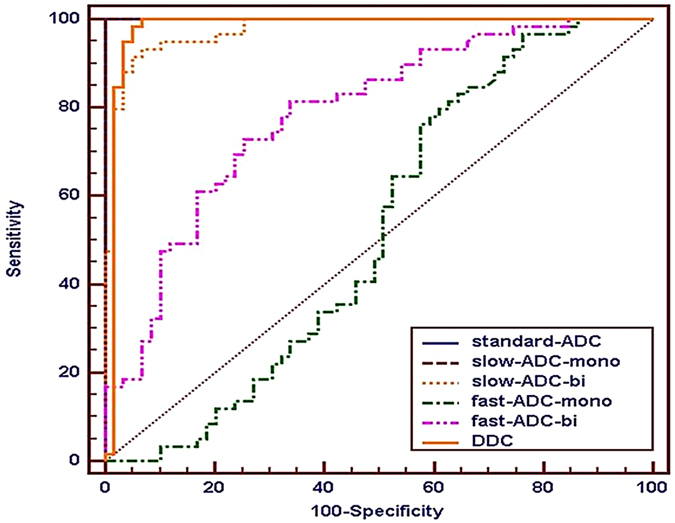
Receiver-operating characteristic curves of diffusion and perfusion coefficients in the differentiation between the lesion and normal rectal wall.

**Figure 5:**
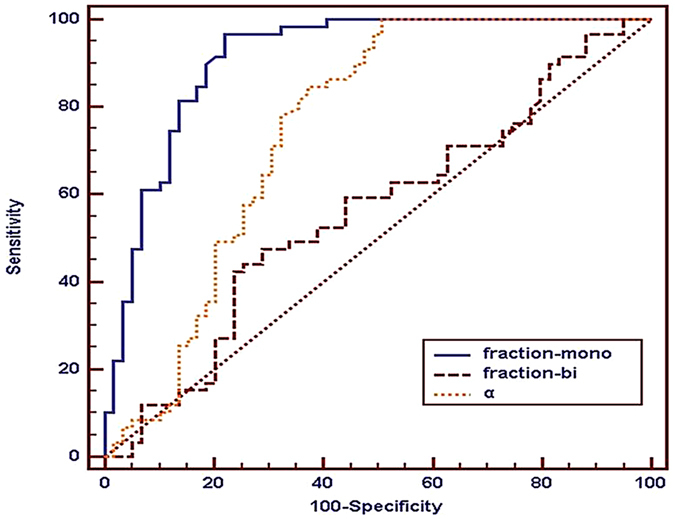
Receiver-operating characteristic curve of Fraction-Mono, Fraction-bi and α in the differentiation between the lesion and the normal rectal tissue.

The area under the curves (AUCs) of Standard-ADC and slow ADC-mono with 100% sensitivity and specificity were bigger than that of other diffusion parameters (slow ADC-bi and DDC). However, there were no statistically significant differences between different diffusion parameters. The AUCs of perfusion parameters (fast ADC-mono and-bi) were significantly smaller than that of diffusion parameters, and the specificity of fast-ADC-mono was only 23.73%. ROC maps were shown in Figs 4 and 5 and the detailed results were in Table 2.

### CNR (contrast to noise ratio) of DWI at different b-values

**Figure 6:**
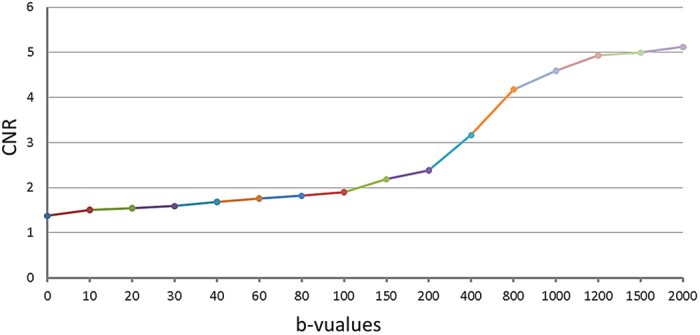
The CNR increased gradually with the growth of b-value till b approaches 1200 s/mm^2^, when the CNR curve reached a relative plateau.

As seen in Fig. 6, the CNR value kept increasing with the growth of b-value till b < 1200 s/mm^2^, when it entered a relative plateau. One-way ANOVA showed statistically significant differences between five CNRs (at b = 800, 1000, 1200, 1500, 2000 s/mm^2^, P < 0.001, Table 3). Multiple comparisons showed no statistically significant differences between CNR at b = 1000 and b = 800, 1200, 1500, 2000 s/mm^2^ (P = 0.389, 0.876, 0.443, and 0.085, respectively, Table 3).

## Discussion

In this study, diffusion weighted signal decay was analyzed using non-Gaussian diffusion models and a Gaussian diffusion model. The result indicated substantial deviation of water diffusivity from mono-exponential attenuation within the applied b-factor range in rectal cancer and normal tissue. SEM and IVIM-mono models achieved a significantly better fit for the tumor than the mono-exponential model, and only SEM was better than the mono-exponential model in relation to the normal rectal wall. This indicated that significantly better fitting of DWI signals could be achieved by non-Gaussian diffusion models, with the exception of the IVIM-bi model, which obtained a comparably good fit in relation to normal tissue but a poor fit in relation to rectal cancer, and SEM was better than IVIM. The result could be explained by the distribution of diffusion-driven displacements being much more complicated in a living body. It is known that the distribution of water molecular displacements obeys Gaussian law under ideal conditions. However, diffusion in tissue is restricted by obstacles such as cell membranes, fibers, macromolecules or electric charges at the proteins or cell membrane surfaces. The molecular displacement distribution then deviates from Gaussian diffusion and this effect can no longer be adequately described by the mono-exponential model on MR imaging^15^.

For the IVIM method, the CVs of perfusion parameters (fast-ADC, f) derived from IVIM-mono and –bi were higher than that of other parameters, which might be attributed to highly disorganized tumor vasculature, complicated tissue characteristics and a varied DW-MRI acquisition protocol^22^,^23^. All CVs of tumor parameters with IVIM-bi were bigger than that of IVIM-mono, which indicated that IVIM-mono may be superior to IVIM-bi. The fast ADC-bi values of the tumor and the normal rectal wall were much bigger than fast ADC-mono values of both, which showed that the IVIM-bi model may overestimate the proportion of perfusion in water diffusivity. Moreover, some studies^21^,^24^,^25^,^26^ found that there was no or weak correlation between fast-ADC and CBF, f value and CBV, which meant that fast-ADC and f value might not represent the true perfusion in the tumor. The AUC of fast-ADC was smaller than that of slow-ADC, which demonstrated the lower diagnostic efficiency of fast-ADC in the discriminating malignant lesions and normal tissue. A recent study^27^ about rectal cancer indicated that fast-ADC and f value were not useful for assessing tumor response to CRT (combined chemotherapy and radiation therapy), which was explained by the poor reproducibility and high uncertainty of fast-ADC and f value^19^,^28^,^29^,^30^. These imperfections would limit the clinical utility of IVIM^31^,^32^,^33^,^34^,^35^. Therefore, Riches *et al*.^31^ suggested that clinical research should focus on slow-ADC within tissues and ignore the perfusion effect, thus to increase the clinical utility of IVIM in diagnosis, prognosis or treatment response. This issue was also confirmed by Nougaret *et al*.^27^, who thought that slow-ADC value was better than ADC value derived from the mono-exponential model in assessing rectal cancer response to CRT.

The extreme heterogeneity of malignant tumor parenchyma would lead to a marked decrease of the diffusion coefficient. Results in the present study demonstrated that rectal cancer showed a significantly lower standard-ADC value, lower slow-ADC values derived from both IVIM-mono and IVIM-bi, and a lower DDC value derived from SEM compared with the normal rectal wall. Slow-ADC value was lower than standard-ADC value of no matter tumor or normal, which could be explained by the contribution of perfusion to the diffusion coefficient being removed. Standard-ADC value had a high diagnostic efficacy (AUC = 1, sensitivity and specificity = 100%) and low CV (8.78%), which should be attributed to the application of the multi b-value (16 b-values). This agreed with Thoeny’s^36^ suggestion that multiple b-values could achieve more reliable results and avoid selection bias even for the perspective of a mono-exponential model. The AUCs of different diffusion coefficient were extremely high and roughly equivalent, and significantly higher than perfusion parameters (fast ADC-mono and-bi) and α value. This meant that diffusion coefficients were the main effective diagnostic parameter in distinguishing lesions from normal tissue. DDC value with high diagnostic efficacy (AUC = 0.980, sensitivity = 98.31%, specificity = 94.92%) in differentiating tumor from normal rectal wall and relatively low CV (16.59%) has not been applied in previous studies of rectal cancer, and SEM exhibited the best fitting results of actual DWI signal in rectal cancer and the normal rectal wall (R^2^ = 0.998, 0.999), therefore, SEM may be a new method for studying rectal cancer.

We found that CNR (tumor-normal) increased gradually with the growth of b-value, and that there were no statistically significant differences between CNR b = 1000 vs b = 800, 1200, 1500, 2000 s/mm^2^ (P = 0.389, 0.876, 0.443, and 0.085). We also know that SNRs would decrease with higher b-value. In order to obtain a better image quality as well as good CNR, b = 1000 s/mm^2^ was suggested as optimal b-value for displaying clearer border between tumor and normal tissue, which we think is helpful for clinical work. When we outlined the tumor on the DWI image with b = 1000 s/mm^2^, we found the T2 shine-through effect and the disturbance of necrosis could be effectively avoided. That was also the reason why ROIs were put on DWI image with b = 1000 s/mm^2^ in this study.

There are three main limitations in this study. Firstly, group discussions for different models in accordance with tumor differentiation did not take place due to the small sample size, especially in relation to well differentiated rectal cancers. In the future, more subjects are needed to be recruited for investigating the capabilities of non-Gaussian models in predicting T-staging, and histological differentiation grades. Secondly, artificial error caused by manual ROIs and motion artifacts caused by long time DWI scanning were inevitable, which would have had an adverse influence on the results. Thirdly, the b-factor applied in this study may not be optimal for all non-Gaussian models and the scanning protocol could also affect the parameter quantification performances for different non-Gaussian models^37^. This needs to be further optimized in order to balance parameter estimation reliability with minimum sampling time.

In conclusion, non-Gaussian diffusion models could assess tissue properties more reasonably than the Gaussian diffusion model with multi-b-values DWI. Based on this explorative study, SEM may be used as a potential optimal model for characterization of rectal cancer, and IVIM-mono may provide a promising functional parameter (slow ADC-mono) for further research in rectal neoplasm.

## Methods

### Patients

The institutional ethical review board of Xijing Hospital approved this study and written informed consent was given by each patient. This study was conducted in accordance with the Declaration of Helsinki. A total of sixty-seven rectal cancer patients were included in this study. Their condition was confirmed by colonoscopy which was followed up by an MR examination, from February 2015 to March 2016. The time interval between MR imaging and surgical treatment was less than two weeks. Five patients with no obvious lesions or minor lesions (area of ROI is less than 100 mm^2^) were excluded. Two patients with neuroendocrine carcinomas and one patient with a melanoma were also excluded. Therefore, fifty-nine rectal adenocarcinoma patients confirmed by postoperative pathology were finally enrolled and analyzed: fourteen poorly differentiated rectal cancers, forty moderately differentiated rectal cancers and five well differentiated rectal cancers.

### MRI protocol

All MR scans were performed on a 3.0 T MR scanner (Discovery MR750, GE Medical Systems, Milwaukee, WI, USA) with an 8-channel phased-array coil. Bowel preparation was generally required before the examination. Rectal MRI sequences included axial T1WI (TR/TE = 487/8), coronal and sagittal T2WI (TR/TE = 7355/136), axial FRFSE T2WI (TR/TE = 7096/133) with small FOV (220 mm), routine DWI (b = 0, 1000 s/mm^2^), and multiple b-value DWI (single-shot SE-EPI diffusion-weighted sequence) with 16 b-values of 0, 10, 20, 30, 40, 60, 80, 100, 150, 200, 400, 800, 1000, 1200,1500, and 2000 s/mm^2^ (TR/TE=4431/71, FOV 260 mm, matrix 128 × 128, slice thickness 5 mm, intersection gap 0.5 mm, NEX 1 to 10 with the increasing of b-values, total DWI scan time 6:34 min).

### Models of DWI

All functional maps of different parameter were post-processed using the MADC programs on an AW 4.6 workstation provided by the manufacturer (GE Healthcare) according to the equations below:

Traditional mono-exponential diffusion model:


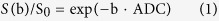


The traditional mono-exponential model provides the apparent diffusion coefficient (ADC) which represents the distribution of diffusion-driven displacements and obeys the Gaussian law. Where S_0_ and S(b) are the signal intensity obtained with the b0 and b1 images. In abdomen MRI, the most common b0 and b1 value is 0 mm^2^/s and 1000 mm^2^/s respectively. In this study, the standard-ADC was investigated instead of conventional ADC. It was also derived from the conventional mono-exponential diffusion model. The only difference between the standard-ADC and the conventional ADC is that standard-ADC is calculated by multi-b-values while the conventional ADC is calculated by only two b-values.

Intra-voxel incoherent motion (IVIM) model^38^:


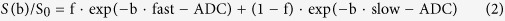


The intra-voxel incoherent motion (IVIM) theory^38^ is based on two separate proton pools that estimate fast and slow diffusion components individually. Where S is the mean signal intensity according to the b-value. The fast-ADC (perfusion coefficient) represents the average blood velocity and mean capillary segment length. The contribution of fast-ADC to the signal attenuation mainly exists at lower b-values (usually, b < 200 s/mm^2^). The f (perfusion fraction) represents the ratio of water movement within capillaries compared with the total volume of water in a voxel. The slow-ADC (diffusion coefficient) represents pure molecular diffusivity where a physiological perfusion effect is excluded^38^,^39^. For the IVIM-mono model, DWI data fitting was performed using the asymptotic fitting method, which provided a more accurate and robust estimation than the full fitting of DWI signals (for IVIM-bi model) to the bi-exponential function^40^. In detail, IVIM-mono was first obtained with a least-square fitting to a mono-exponential function by using the data points at b-values over 200 s/mm^2^. The fitted curve was then extrapolated to obtain an intercept at b = 0. The ratio between this intercept and the DWI data point at b = 0 gave an estimate of f. Finally, the obtained slow-ADC and f were substituted into “equation (2)” and non-linear least-square fitted against all b-factors to estimate fast-ADC^16^. As for the IVIM-bi model, all 16 b-values were used as input data for the IVIM parameters. The high b-values generate slow-ADC at first, and the low b-values will yield fast-ADC and f at the same time after removing the effects of slow-ADC^17^.

Stretched exponential model (SEM)^41^:


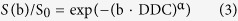


The stretched-exponential function considered^41^ that each voxel consists of a collection of protons with a distribution of (apparent) diffusion coefficients, but does not require an assumption about the shape of the distribution of apparent diffusion rates or the number of discrete apparent diffusion rates present. This model provides an approximate, complete and accurate empiric description of tissue water diffusion^42^. It can be simply understood as an adjustment to the mono-exponential model by α^43^ which was used to characterize heterogeneity of the diffusion signal. The Eq. 3 will be equivalent to “equation (1)” when α = 1, which indicates that the distribution of water molecule diffusion in pure water obeys the Gaussian law and will no longer obey Gaussian law with increased heterogeneity of tissue. Where, DDC is the distributed diffusion coefficient, α is the heterogeneity index (0 ≤ α ≤ 1).

### ROI setting

**Figure 7:**
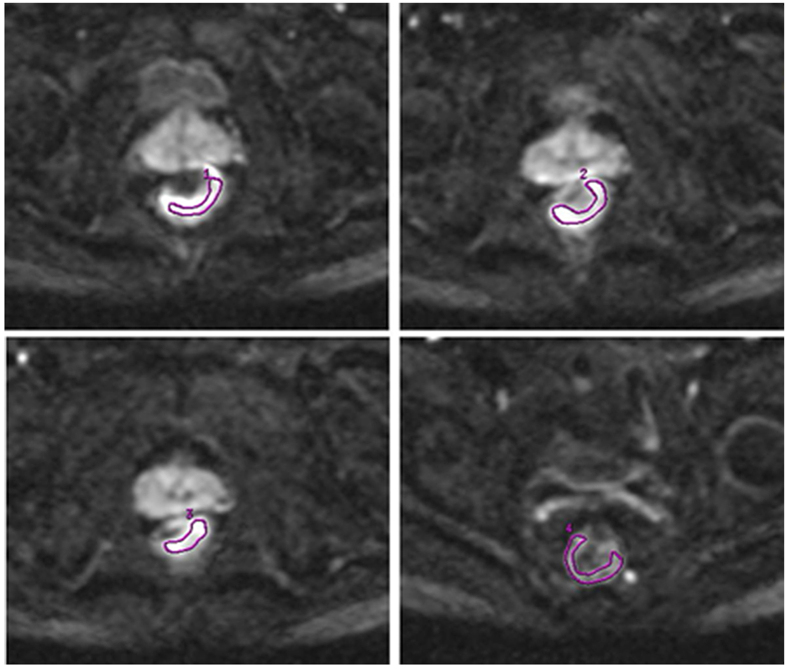
ROIs (1–3) were drawn on three consecutive slices with three of the largest cross-sectional areas of rectal tumor. The final value of the tumor’s ROI was the average of ROIs (1–3). ROI 4 (normal rectum) was drawn on an adjacent slice to the tumor. All ROIs were manually drawn on the DWI image with b = 1000 s/mm^2^ and the area of each ROI was not less than 100 mm^2^.

Region-of-interests (ROIs) for each rectal tumor were manually drawn along the margin of the tumor on three consecutive slices with three of the largest cross-sectional areas of tumor on axial DWI images with b = 1000 s/mm^2^ by two expert radiologists, each with more than 10 years of experience, and who were unaware of the pathologic diagnosis. The ROI should include the solid part of the tumor, as much as possible, and exclude necrotic signal areas. ROI for the normal rectal wall was put on an adjacent slice to the tumor, which at least covered a half circle of the normal rectal wall. The distance from the tumor exceeded 5 mm. Each ROI’s area was no less than 100 mm^2^. A well-matched copy of the ROI is automatically and synchronously generated and appears on each functional mapping of MRI parameters on corresponding locations by the built-in software (MADC programs on an AW 4.6, GE Healthcare, Milwaukee, WI). The ROI setting is shown in Fig. 7. Each functional parameter’s result and DWI signals for tumor and normal tissue were respectively calculated by the average value of three ROIs (1, 2, and 3) on tumor area and the mean value of a single ROI (4) on normal tissue.

### CNR (contrast to noise ratio) of DWI at different b-values

All CNRs (tumor vs normal) of the 16 b-values of DWI were calculated according to the equation below:


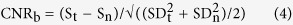


Where, S_t_ and S_n_ are signal intensities of tumor and the normal rectal wall within the ROIs, respectively. SD_t_ and SD_n_ are standard deviations of tumor signal intensity and normal rectal wall signal intensity. CNR_b_ is the CNR of DWI image at a given b-value.

### Measurement repeatability test

The measurement reliability of all parameters between inter-observers was evaluated by using the intraclass correlation coefficient (ICC). The ICC was regarded as good when it was >0.75, as moderate when it was ≥0.4 but <0.75, and as poor if it was <0.4.

### Statistical Analysis

Statistical analyses were performed using SPSS version 19.0. P values < 0.05 were considered significant for all tests. The association between S(b)/S_0_ and b-values was analyzed under four models, namely Gaussian diffusion model (mono-exponential model), SEM, IVIM-mono model and IVIM-bi model. Adjusted coefficient of determinant (R^2^) was calculated to evaluate the goodness-of-fit for each model. R^2^ < 0.8 as considered as the fitting result for signal attenuation trend was poor. Furthermore, F analysis was applied to test the significance of R^2^. A Student’s Test was utilized to compare the tumor with the normal rectal wall for each parameter. One-way ANOVA was used for comparison between the five CNR groups (at b = 800, 1000, 1200, 1500, 2000 s/mm^2^), and a Bonferroni test was applied to make multiple comparisons between CNRs at the five b-values. We adopted receiver-operating characteristic (ROC) Analysis to test the value of functional parameters in differentiating the tumor from normal tissue, and all subjects were involved. ROC analysis was performed using MedCalc version 12.3.

## Additional Information

**How to cite this article**: Zhang, G. *et al*. Comparison of non-Gaussian and Gaussian diffusion models of diffusion weighted imaging of rectal cancer at 3.0 T MRI. *Sci. Rep.* **6**, 38782; doi: 10.1038/srep38782 (2016).

**Publisher's note:** Springer Nature remains neutral with regard to jurisdictional claims in published maps and institutional affiliations.

## Figures and Tables

**Table 1:** Comparisons of different parameters in tumor and the normal rectal wall.

Parameters	*Tumor*	*Normal*	*CV* (*tumor*)	*t*	P*
Standard-ADC (×10^−3^ mm^2^/s)	0.820 ± 0.072	1.414 ± 0.132	8.78%	30.787	<0.001
Slow ADC-Mono (×10^−3^ mm^2^/s)	0.609 ± 0.049	1.080 ± 0.095	8.05%	35.213	<0.001
Fast ADC-Mono (×10^−3^ mm^2^/s)	10.692 ± 4.003	12.366 ± 7.185	37.44%	1.42	0.161
f-Mono	0.278 ± 0.044	0.414 ± 0.094	15.83%	9.913	<0.001
Slow ADC-bi (×10^−3^ mm^2^/s)	0.538 ± 0.167	1.155 ± 0.259	31.04%	14.866	<0.001
Fast ADC- bi (×10^−3^ mm^2^/s)	42.891 ± 19.588	70.358 ± 28.677	45.67%	6.104	<0.001
f- bi	0.318 ± 0.069	0.306 ± 0.085	21.70%	0.812	0.42
α value	0.724 ± 0.043	0.781 ± 0.074	5.94%	4.704	<0.001
DDC (×10^−3^ mm^2^/s)	0.850 ± 0.141	1.722 ± 0.415	16.59%	15.707	<0.001

All values are expressed as mean ± SD. f-Mono, f-bi and α have no unit. DDC (distributed diffusion coefficient), α (alpha, the heterogeneity of intra-voxel diffusion), CV (coefficient of variation).

^*^The comparison between tumor and normal rectal wall for every parameter was used by the Student’s Test. P < 0.05 was considered significant.

**Table 2:** Optimal Cutoff Values for Differentiation between the lesion and the normal rectal wall.

Parameters	Cutoff value	AUC (95%CI)	Sensitivity	specificity	*P* value
Standard-ADC(×10^−3^ mm^2^/s)	0.976	1.000 (0.969–1.000)	100%	100%	1.000^a^
Slow ADC-Mono(×10^−3^ mm^2^/s)	0.773	1.000 (0.969–1.000)	100%	100%	0.037^b^
Fast ADC-Mono (×10^−3^ mm^2^/s)	18.267	0.520 (0.426–0.613)	96.61%	23.73%	0.226^c^
Fraction-Mono	0.348	0.911 (0.845–0.956)	96.61	77.97	0.037^d^
Slow ADC-bi (×10^−3^ mm^2^/s)	0.729	0.975 (0.928–0.995)	91.53%	94.92	0.226^e^
Fast ADC-bi(×10^−3^ mm^2^/s)	60.733	0.786 (0.700–0.856)	81.36%	66.10%	0.827 ^f^
Fraction-bi	0.32	0.558 (0.464–0.649)	47.46	71.19	
α value	0.806	0.751 (0.663–0.826)	100%	49.15	
DDC (×10^−3^ mm^2^/s)	1.124	0.980 (0.935–0.997)	98.31%	94.92%	

AUC (area under the curve), CI (confidence interval), superscript letters of ^a^(Standard ADC vs Slow ADC-Mono), ^b^(Standard ADC vs Slow ADC-bi), ^c^(Standard ADC vs DDC), ^d^(Slow ADC-Mono vs Slow ADC-bi), ^e^(Slow ADC-Mono vs DDC), ^f^(Slow ADC –bi vs DDC). P values < (0.05/6) were considered significant after Bonferroni correction.

**Table 3:** CNRs (b = 800, 1000, 1200, 1500, 2000 s/mm^2^).

b-value	CNR	F	*P*	*P1*/*800*	*P2*/*1000*	*P3*/*1200*	*P4*/*1500*	*P5*/*2000*
800	4.17 ± 1.08	7.314	0.001*	—	0.389	0.002	0.001	0.001
1000	4.59 ± 1.09	—	—	0.389	—	0.876	0.443	0.085
1200	4.93 ± 1.10	—	—	0.002	0.876	—	0.999	0.999
1500	4.99 ± 1.08	—	—	0.001	0.443	0.999	—	0.999
2000	5.12 ± 1.07	—	—	0.001	0.085	0.999	0.999	—

^*^One-way analysis of variance, *P1*–*5* Bonferroni test was used for multiple comparisons between CNRs at five b-values. — means no value.
